# Nanosecond laser coloration on stainless steel surface

**DOI:** 10.1038/s41598-017-07373-8

**Published:** 2017-08-02

**Authors:** Yan Lu, Xinying Shi, Zhongjia Huang, Taohai Li, Meng Zhang, Jakub Czajkowski, Tapio Fabritius, Marko Huttula, Wei Cao

**Affiliations:** 1 School of Materials Science and Engineering, Henan University of Science and Technology, Luoyang, 471023 China; 2 Nano and Molecular Systems Research Unit, University of Oulu, P.O. Box 3000, FIN-90014 Oulu, Finland; 3 School of Mechanical and Automotive Engineering, Anhui Polytechnic University, Wuhu, 241000 China; 4 College of Chemistry, Key Lab of Environment Friendly Chemistry and Application in Ministry of Education, Xiangtan University, Yuhu District, Xiangtan, 411105 China; 5 Department of Physics, East China University of Science and Technology, Meilong Road 130, Shanghai, 200237 China; 6 Optoelectronics and Measurement Techniques Research Unit, University of Oulu, P.O. Box 4500, FIN-90014 Oulu, Finland

## Abstract

In this work, we present laser coloration on 304 stainless steel using nanosecond laser. Surface modifications are tuned by adjusting laser parameters of scanning speed, repetition rate, and pulse width. A comprehensive study of the physical mechanism leading to the appearance is presented. Microscopic patterns are measured and employed as input to simulate light-matter interferences, while chemical states and crystal structures of composites to figure out intrinsic colors. Quantitative analysis clarifies the final colors and RGB values are the combinations of structural colors and intrinsic colors from the oxidized pigments, with the latter dominating. Therefore, the engineering and scientific insights of nanosecond laser coloration highlight large-scale utilization of the present route for colorful and resistant steels.

## Introduction

Beautifying and passivating metal surfaces is highly time, energy and material consuming process in modern industry. Recently developed laser coloration has been achieved on stainless steel but it has been greatly limited for expensive sources. In addition, the lack of physical knowledge of the mechanism beyond color appearance and performance have prevented the boom of the promising technique. The colorful stainless steel has drawn much attention due to its excellent performance in architectural and decorative applications^1^. Conventional chemical erosion and electrochemical methods^2, 3^ to introduce colors on steel surface are gradually abandoned due to the environmental pollution problems. The hunt for a facile and costless method of fabricating colored stainless steel have been active for years by both industrial and academic fields. One feasible route was brought about by femtosecond laser technique, which has been applied successfully in preparing black silicon, iridescent aluminum and blue titanium surfaces^4–6^. The femtosecond laser with high photon energy is efficient at producing laser-induced periodic surface structure (LIPSS) on steel surface^7^. Such LIPSS surface shows enhanced hydrophobic and corrosion resistant abilities, and is also endowed with abundant colors^8–10^. However, high-cost femtosecond laser is not well suitable for industrial production, while the alternative nanosecond laser was believed that it could only produce limited colors species^11^. Current researches of these routes mainly stay at the engineering level, just producing series of colors by tuning laser parameters^12, 13^.

Despite the limitation arisen from materials engineering, mechanisms leading to the surface coloration remain unclear. Coloration of femtosecond laser markings is primarily attributed to the structure color effect, but as for markings fabricated by nanosecond laser, such light-matter interference will be extremely decreased because the surface structure is in a much larger scale than the wavelength of visible light. As for the route employing nanosecond sources, although the color range has been extended gradually^14^, the coloration mechanism has not been sufficiently studied. Several reports referred to the intrinsic colors of the metal oxides in the laser markings^12–14^, but lack of further approval and discussion. Contributions from different pigments or interferences to RGB values on the decorated steels are not quantified to the best of our knowledge. Therefore, understanding the impact of laser-induced structural and compositional variations on the apparent colors becomes crucial to promote the nanosecond laser marking technique in large-scale industrial applications.

In this work, we carried out a novel study to explore mechanism of nanosecond laser coloration on 304 stainless steel surface, and quantitatively matched the RGB values on the colored steels to the corresponding abundances of oxides determined at atomic level. Square markings with straw-yellow, cornflower-blue and fuchsia colors were prepared under a master-oscillator-power-amplifier (MOPA) laser system in ambient air at room temperature. Structural color effect was studied by both simulation of the thin film interference and measurements of optical reflectance properties. Meanwhile, elemental and chemical states analysis of the marking samples was carried out through X-ray photoelectron spectroscope (XPS). The compositional color contribution was thus quantitatively discussed.

## Results

### Color information

Various colors were formed on the mechanically polished 304 steel surfaces via the low-cost and fast nanosecond pulse laser coloration route. A typical radiation time is around 1 minute to color each square with an area of 5 × 5 mm^2^. Different colors were reached via semi-empirically varying laser scanning speed, repetition rate, and pulse width. The relations between nanosecond laser parameters and final surface performance have been discussed elsewhere^12–15^, yet out of the main focus of the present work.

**Figure 1:**
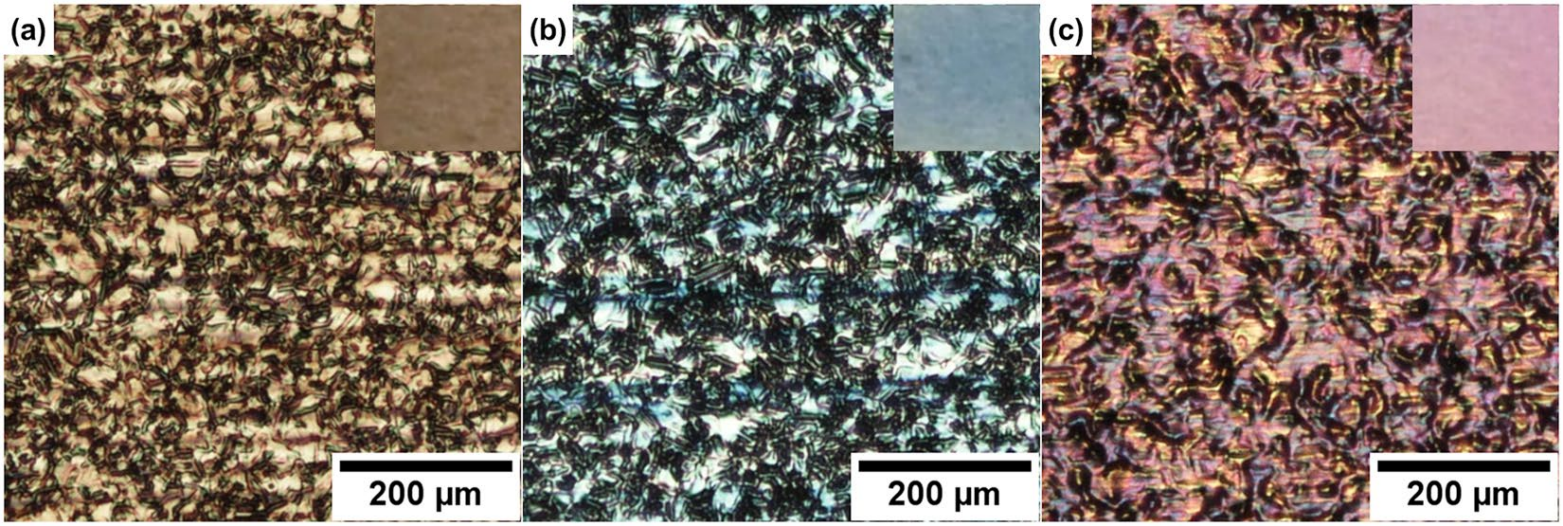
Optical microscopy images of the colorized surface. (**a**) Sample 1, straw-yellow, (**b**) Sample 2, cornflower-blue, (**c**) Sample 3, fuchsia. Photos of insets were taken by digital camera.

Color information of the laser markings was obtained through polarized light microscope without filters. During the measurements, the incident white light beam was kept perpendicular to the sample surface. As shown in Fig. 1, three colorized samples were prepared, displaying straw-yellow, cornflower-blue and fuchsia colors, respectively. The insets were taken by digital camera with tilted directions (raw photo shown in Supplementary Fig. 1), appearing slightly different colors. To quantitatively describe color information, the commonly used RGB color model is presented here. The RGB model is built following the way in which cone cells in human retina perceive red, green and blue colors^16^, and all the three color components vary from 0 ~ 255. A larger component value indicates a higher brightness and saturation. In order to get the average RGB values of each photo, the red, green and blue values of each pixel were counted and averaged. Table 1 indeed shows that all the generated colors have relatively low RGB values, or in other words, colors are not fully saturated.

**Table 1:** Color information of the laser markings.

	Red	Green	Blue
Sample 1	123	100	75
Sample 2	95	106	101
Sample 3	123	91	83

### Optical reflectance

**Figure 2:**
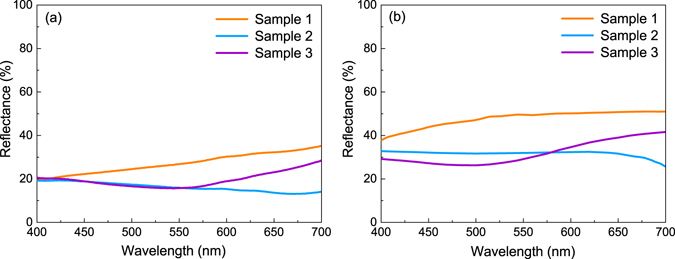
Optical reflectance property measured with incident angles of: (**a**) 30°, (**b**) 70°.

Optical reflectance was measured at the incident angle of 30° and 70°, respectively. With Xenon lamp as the light source, the incident light spectrum agreed well with the solar spectrum in the visible light range^17^. In both Fig. 2a and b, low reflectance values were recorded, yet it is noteworthy that the reflectance increased slightly with the incident angles. This is the characteristic behavior of structural color effect. In general, the presented colors vary in a limited scope. Differences between Fig. 1a~c and the insets suggest a weak influence on the final colors from the aspect of light-matter scattering. Among the three samples, sample 1 exhibits the highest reflectance. Meanwhile, a rise of ~20% in the reflectance indicates that sample 1 is more influenced by the structure color. For each sample, the reflectance roughly increases along with the light wavelength, in accordance with results in previous research^18^.

### Contribution of structural color

**Figure 3:**
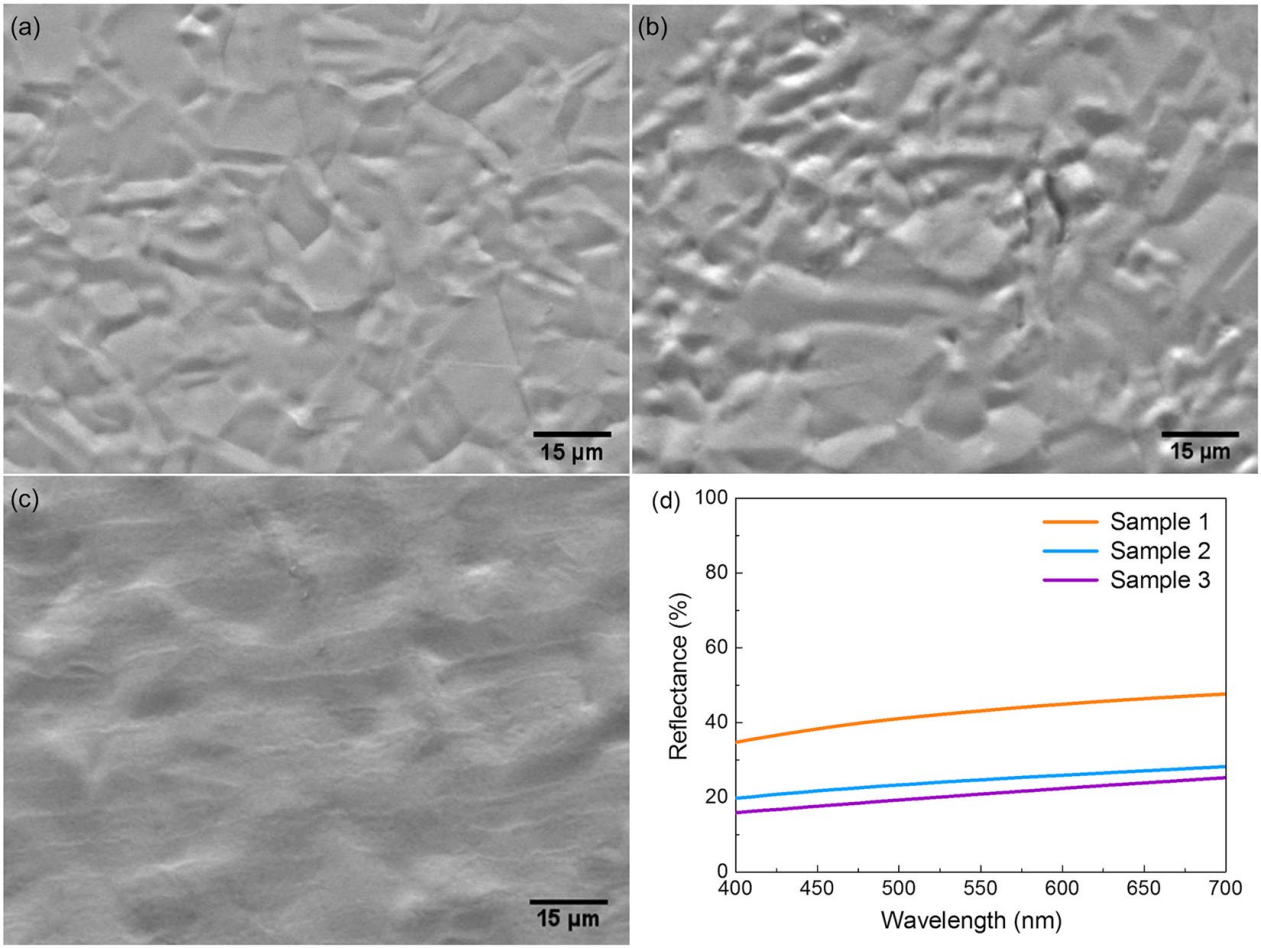
Morphologies of laser marking and the simulation results of reflectance. (**a**)~(**c**) SEM images of sample 1~3, respectively. (**d**) Simulation results of optical reflectance.

To clarify the light-matter interaction, it is essential to acquire the information of morphology and thickness of the laser-induced layer on steel surface. Sample morphologies depicted in Fig. 3a–c show that the surfaces are basically composed of irregular micro-wrinkles with lateral dimensions in 10 µm scale, much larger than the wavelength of visible light. Thus, possibility of light interference with the microstructure is rather low^19^. Although sample 1 and 2 show distinct colors, their microstructures are quite similar except that the wrinkles in sample 1 are larger and arranged more sparsely. Short-order periodic grooves appear in sample 2 (Fig. 3b), but their influence on the coloration can be still neglected due to the lack of quantity. In Fig. 3c, there are uniformly arranged gratings. However, the periodic length is around 8 ~ 12 µm, far from the coherent length to interfere with visible light. Therefore, the structural color effect may only originate from the light interference with the laser-generated film.

The film on top of steel surface was formed during laser treatment, and its thickness can be estimated through surface profile measurements. The layer thickness of sample 2 is ~300 nm, while the other two are approximately 800 nm (Supplementary Fig. 2). As an alternative non-destructive technique, ellipsometry method^20^ was also employed to double-check the thickness. For sample 2, the ellipsometry method gave the thickness of 314.4 ± 37.6 nm (Supplementary Fig. 3), which is very close to the surface profiler result (300 nm). However, such a method could not be accomplished for sample 1 and sample 3, even with the AutoRetarder which allows depolarization for surface of certain roughness. It might be due to rather high roughness and limited region of the laser induced films, which are beyond the applicability of the surface-sensitive ellipsometry method^21^. In this case, we carried out cross-sectional scanning electron microscope (SEM) measurement in order to directly obtain the film thicknesses (Supplementary Fig. 4). Values of 764.1 ± 31.7 nm, 338.1 ± 20.9 nm and 807.6 ± 54.2 nm were found for sample 1–3, respectively, in good agreement with the optical profilometer results.

Simulation work was carried out to evaluate the reflective ability coming from thin-film interference. Smooth film models were built, and the morphology information on the surface was discarded. The layer thickness was set according to the surface profile results, while incident light set perpendicularly. The refractive indices and extinction coefficients were set based on ellipsometry measurement results. The simulated results in Fig. 3d have the same trend of wavelength dependence as the experimental determinations in Fig. 2. However, discrepancies of ~10% have been found between the calculated and measured reflectance for sample 2 and sample 3. This can be attributed to typical simplifications of theoretical models and homogeneity of materials employed in the simulations^22, 23^. Indeed, even for a surface composed of single element (e.g., bare silicon surface), theoretical results^22^ can still vary more than 10% against experimental ones^24^. In spite of the discrepancies, no obvious peaks can be observed in the reflectance curves. Thus, structural coloration does not dominate final colors on the nanosecond laser marked surfaces.

### Quantification of compositions

Quantitative analysis of surface compositions is the prerequisite to evaluate the composition based coloration mechanism. By the energy dispersive spectroscopy (EDS), we roughly examined the elemental information of the samples. Fe, Cr, Ni and Mn were the primary elements, and more Cr was detected in sample 1 (see Supplementary Table 1). Since the detection depth of EDS (~1 µm)^25^ is much larger than the film thickness, EDS result was mixed up with elemental information coming from substrates, making it inappropriate to analyze the thin films on the surface.

**Figure 4:**
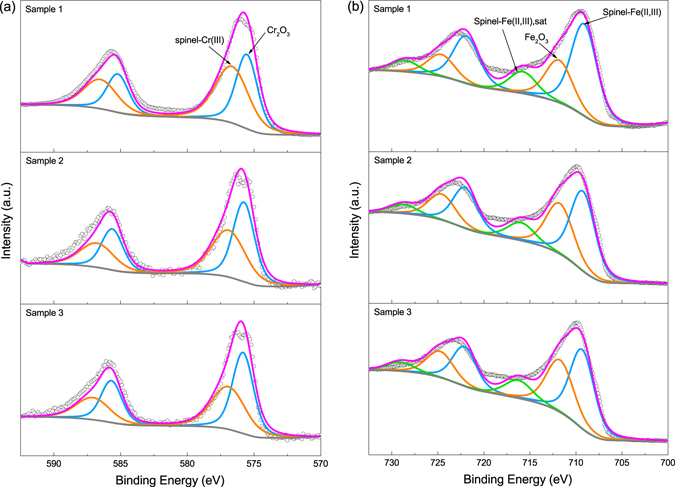
XPS spectra of: (**a**) Cr 2*p*, (**b**) Fe 2*p*. In each panel, the spectra of the three samples are illustrated. Each pair of doublet peaks were illustrated with identical color. The scatter plots are experimental results, while the magenta and grey lines represent the fitting envelopes and backgrounds, respectively.

XPS is a surface sensitive technique and thus available to focus on the thin markings. Figure 4 shows the XPS spectra of Cr 2*p*, Fe 2*p* and the details of peak fitting are listed in Supplementary Table 2. For Cr 2*p* spectra, two groups of peaks were identified in Fig. 4a, which were Cr_2_O_3_ at ~576.5 eV and a spinel structure at ~575.4 eV, respectively^26, 27^. The typical spinel structure is XY_2_O_4_, where X and Y represent ions in +2 and +3 valence^28^. In this work, its form can be specified as (Mn^2+^_x1_Ni^2+^_x2_Fe^2+^_x3_)(Fe^3+^_x4_Cr^3+^_x5_)O_4_, where x_1_ + x_2_ + x_3_ = 1 and x_4_ + x_5_ = 2. In contrast, the spinel peaks disappeared on the spectra of steel substrates, and chromium of metal form was detected (Supplementary Fig. 5). Similar results are also found in Fig. 4b. The spinel compound and Fe_2_O_3_ were identified. As a typical feature for Fe 2*p* spectra of stainless steel, the pair of satellite peaks of spinel compound were also specified. It suggests that the spinel structure was formed during the laser treatment process.

Indeed, native passivation layer on stainless steel surface is mainly composed of Cr_2_O_3_, Fe_3_O_4_ and Fe_2_O_3_^29^. Alloy elements were promoted to disperse from the substrate to the passivation layer due to the heat effect of pulsed laser, and they could be more easily oxidized by oxygen. Oxygen partial pressure was therefore reduced, producing a reductive atmosphere in local region. In this case, Cr and Mn have the priority to react with the remaining oxygen and form their metal oxides. After that Fe was also oxidized to FeO, meanwhile, laser beam moved away and the temperature as well as the reaction rate dropped consequently. Oxygen partial pressure rose, which facilitated the simultaneous oxidization of Fe, Ni and the metal oxides. The spinel compound was then formed. It was also possible for Cr^3+^ substituted by Fe^3+^, making the spinel structure even more complicated^30^.

It is noteworthy that very small amount of Ni was detected although there was 8~11% in the commercial 304 steel substrate (see Supplementary Table 2 and Supplementary Fig. 6). Ni is nearly absent in the native passivation layer, while that in the substrate usually completely dissolved in γ–Fe, making it difficult to escape^31^. The EDS results indicated more than 6% of Ni, most of which should come from the substrates.

## Discussion

Obvious discrepancies between the real colors on the surfaces and optical determinations demand further investigations of the color origins of the laser treated steel surface. It has been noticed the metal oxides, especially well crystalized ones, can also act as stable pigments. Thus, in the following, we carefully examined chemical components on the surfaces, and estimated their contributions to the RGB values. The XPS fitting results provide quantitative information of each element in the sample. For Cr 2*p*, Fe 2*p*, Mn 2*p* and Ni 2*p* spectra, each spectrum was fitted with two components: the metal oxide and the spinel compound. Based on the atomic percentage of the metal ions (in metal oxides), the relative contents of their metal oxides could be easily obtained by dividing the subscript numbers. That is,

1 $${C}_{MO}=\frac{{C}_{ion}}{{n}_{i}}$$

where *C*_*MO*_, *C*_*ion*_ and *n*_*i*_ are the relative contents of metal oxides, the elemental percentage of ions in the metal oxides, and their subscript numbers, respectively. Similarly for the ions in the spinel (Mn^2+^_x1_Ni^2+^_x2_Fe^2+^_x3_)(Fe^3+^_x4_Cr^3+^_x5_)O_4_, divided by the corresponding subscripts, the relative content of spinel could be calculated through any of the metal ions.

2 $${C}_{spinel}=\frac{{C}_{s-ion}}{{x}_{i}}$$

where *C*_*spinel*_, *C*_*s-ion*_ and *x*_*i*_ are the relative content of the spinel, the elemental percentage of metal ions in the spinel and their subscript numbers in the molecular formula. Through equations (1) and (2), we can calculate the relative contents of each metal oxide and the spinel, and also the ratios of each ion in the spinel. The relative contents of all the metal oxides and spinel compounds were normalized and tabulated in Table 2. The quantified molecular formula of the spinels are (Mn^2+^_0.12_Fe^2+^_0.88_)(Fe^3+^_1.12_Cr^3+^_0.88_)O_4_, (Mn^2+^_0.07_Ni^2+^_0.09_Fe^2+^_0.84_)(Fe^3+^_1.82_Cr^3+^_0.18_)O_4_ and (Mn^2+^_0.07_Ni^2+^_0.04_Fe^2+^_0.89_)(Fe^3+^_1.80_Cr^3+^_0.20_)O_4_ for samples 1–3, respectively.

**Table 2:**
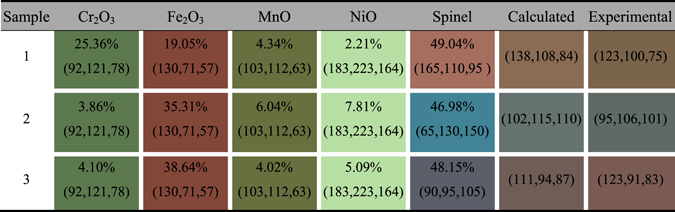
Contents and color information of the compositions, and the calculated and measured RGB values of each sample. The percentage numbers show the contents of the composition. Each cell is shaded with its own RGB values.

Compositions in the laser marking film, say the metal oxides and the spinel compound, are natural pigments presenting distinct colors. RGB values of metal oxides were set according to previous studies of their pure form^32, 33^. However, it becomes complicated when figuring out the color of the spinel. Its color is highly influenced by Cr, Fe elements, yet unfortunately affirmative relationships between the ratios and colors remain unknown. What’s more, the Fe^2+^ and Fe^3+^ have different influence to the colors, and colors change drastically even with a small amount variation. Therefore we propose that the metal oxides and the spinel contribute their own color (in RGB values) by a factor of their relative contents, while the color of the spinel is qualitatively dominated by the existing Fe^2+^, Fe^3+^ and Cr^3+^. Fe^2+^ in the spinel results in a blue color species, varying among grey-blue, violet-blue and even dark green^34^. In contrast, enrichment of Fe^3+^ presents dark brown, while small amount of Fe^3+^ makes the color prone to green or even blue-green^35^ when Fe^2+^ is oxidized to Fe^3+^. On the other hand, Cr^3+^ is the key factor of red appearance, which usually give red or pale pink colors to the spinel^36^. The joint effect of Fe^2+^ and Cr^3+^ then produce colors in violet-red or dark violet, but the color will tend to orange when Fe^3+^ replace part of the Cr^3+^. The effect of Mn^2+^ and Ni^2+^ is not significant due to the very small amount as given by the EDS and XPS results. Mn^2+^ limitedly enhances the saturation of blue color and thus make it close to yellow^37^, while the existence of Ni^2+^ usually produces yellow-green color which is similar to the intrinsic color of NiO^38^. In general, the spinel presents brown color when Fe^2+^, Fe^3+^ and Cr^3+^ exist simultaneously. Considering the specific amount of metal ions in each sample, we are able to set initial RGB values for the spinels. For sample 1, Fe^2+^, Fe^3+^ and Cr^3+^ have almost the same amount, and much of the Cr^3+^ ions entered the center of spinel octahedron, making a reddish brown color. Meanwhile, Fe^3+^ raised the green value, and Mn^2+^ improved the color saturation and mainly increased blue and green values. Thus spinel color could be revised from standard reddish brown (165, 42, 42) to (165, 110, 95). In sample 2 and 3, the amount of Cr^3+^ greatly dropped so that the red values would be much smaller than in sample 1. Compared to sample 3, there are more Fe^3+^ in sample 2 which make a higher saturation of blue. It also contains relatively more Ni^2+^, tending the color closer to green. The spinel color then was set as (65, 130, 150). Fe^3+^ are two times more than Fe^2+^ in sample 3, and it usually presents dark grey (105, 105, 105) in this case. With more Cr^3+^ and less Ni^2+^ than in sample 2, red value would be larger and green value became smaller. (90, 95, 105) should be a reasonable value for the spinel in this sample.

With the contents as well as the RGB values of metal oxides and the spinel, RGB values of each sample can be calculated by the product of its color matrix and content matrix,

3 $$(\begin{array}{c}R\\ G\\ B\end{array})=(\begin{array}{c}\begin{array}{ccc}\begin{array}{cc}\begin{array}{cc}{R}_{1} & {R}_{2}\end{array} & {R}_{3}\end{array} & {R}_{4} & {R}_{5}\end{array}\\ \begin{array}{cccc}{G}_{1} & {G}_{2} & \begin{array}{cc}{G}_{3} & {G}_{4}\end{array} & {G}_{5}\end{array}\\ \begin{array}{cccc}\begin{array}{cc}{B}_{1} & {B}_{2}\end{array} & {B}_{3} & {B}_{4} & {B}_{5}\end{array}\end{array})(\begin{array}{c}{C}_{1}\\ {C}_{2}\\ {C}_{3}\\ {C}_{4}\\ {C}_{5}\end{array})$$

where *R*_*i*_, *G*_*i*_, *B*_*i*_ are the red, green and blue values of Cr_2_O_3_, Fe_2_O_3_, MnO, NiO and the spinel compound, respectively; *C*_*i*_ represents the contents of those compositions. The calculated RGB values agree well with the experimental results (see Table 2). For each sample, both the calculated and measured RGB results are within the same color species although minor differences exist. Such difference may come from the specification of initial spinel colors, but the structural color effect should be also accounted. Despite the surface microstructure of the laser markings, the interference between the thin film and visible light contributes to the final colors depending on its thickness.

In conclusion, we treated the surface of 304 stainless steel with nanosecond laser and studied the coloration mechanism. Different from the femtosecond laser source, nanosecond laser fabricated surface structures in ~20 µm scale, far from the range of visible light wavelength. Therefore the structural color effect was quite weak and the colors of the laser markings were dominantly contributed by the colorful metal oxides and spinel compound. The contribution should be weighted by the relative content of each components. Such findings would be beneficial to the application of low-cost nanosecond laser in surface treatment of steel and other metallic materials.

## Methods

### Preparation

In this work, the commercial AISI 304 stainless steel was used as the substrate (18~20% Cr, 8 ~ 11% Ni, ≤2% Mn and ≤1% Si) with the thickness of 1 mm. Mechanical polishing was applied before laser treatment in order to produce a smooth surface, and the samples were then cleaned with ethanol. The laser treatment was carried out under a nanosecond MF20-E-A fiber laser marking machine in ambient air at room temperature at Han’s Laser Inc., China. The output power was set as 20 W, generating pulsed laser beam with the central wavelength of 1064 nm. During the laser marking process, the maximum pulse energy was 1.10 mJ at the pulse repetition rate of 45–500 kHz and the scanning speed of 100 mm/s–1300 mm/s, while the pulse width was tuned from 4 ns to 260 ns. Scanning steps varied during the laser treatment.

### Characterizations

Polarized light microscope (Nikon Eclipse LV100DA-U) was employed to acquire the color information of the marked steel surface. The light source is within visible wavelength range without optical filters. Low magnification was adopted so that the colorized surface could be recorded in a relatively large scale. Optical properties including reflectance, refractive index and extinction coefficient were measured by an UVISEL-VASE Horiba Jobin-Yvon ellipsometer with a fixed incident angle of 70°. Reflectance behavior was also taken by an UV-vis-NIR Varian Cary 500 spectrophotometer. For the purpose of comparative analysis, the incident angle of the spectrophotometer was set as 30° degree.

The microstructure morphology was characterized by a Zeiss ULTRA plus FESEM. Surface roughness and film thickness were measured by optical profilometer (Bruker ContourGT-K). Ellipsometer (VASE) and cross-sectional SEM were also applied to double-check the thickness. During ellipsometry measurements, the polarization orientation (Ψ) and polarization phase (Δ) were measured with the AutoRetarder. Regression analysis was used to match the experimental data and calculate the thickness. For cross-sectional SEM measurements, the laser marking samples were cut and inlaid with epoxy resin. The image quality was limited due to the weak conductivity of the metal oxides in the laser marking film.

We roughly examined the element contents of each laser marking through EDS. A Thermo Fisher Scientific ESCALAB 250Xi XPS spectrometer was used for detailed analysis of the final chemical states. All XPS spectra were calibrated with C 1 *s* (248.8 eV) and then fitted.

### Simulation

Numerical simulation of light scattering was carried out in the finite differential time domain (FDTD). The OptiFDTD software (free version) was employed here, and the simulations were based on the space and time partial derivatives of discrete time-dependent Maxwell’s equations. From the experimental point of view, the lateral dimensions of surface morphologies have weak interference with visible light, and this was also approved by series of simulation tests. Therefore, models of laser markings were established in thin film interference mode with smooth surface in order to improve the simulation speed. In the two-layer models, the lower layer was stainless steel substrate and the upper one was the laser marking film. The refractive indices of these two layers were defined according to the experimental results. In the simulations, we employed the refractive indices at the wavelength of 550 nm which were recommended by the OptiFDTD software. These are, *n*_1_ = 1.104 + 1.607*i*, *n*_2_ = 2.198–1.045*i*, *n*_3_ = 1.929 + 0.775*i*, *n*_4_ = 2.586 + 2.413*i* for the laser marking films in samples 1–3 and the steel substrates, respectively. The thickness of steel substrate was fixed as 1 µm, while the values were 800 nm, 300 nm and 800 nm for samples 1–3, respectively. Perpendicular input plane light source was set up as Gaussian modulated continuous wave with wavelength from 400 nm to 700 nm. The boundary conditions in X and Y directions were set as Periodic Boundary Condition (PBC), while Anisotropic Perfectly Matched Layers (APML) in Z direction. To receive reflective information, the observation points were set directly above the models.

### Data Availability

The datasets generated during and/or analysed during the current study are available from the corresponding author on reasonable request.

## Electronic supplementary material

Supplementary information
