# Supplementary material for: Nanosecond laser coloration on stainless steel surface
Source: Sci Rep. 2017 Aug 2;7:7092. doi: 10.1038/s41598-017-07373-8 (PMC5541041; doi:10.1038/s41598-017-07373-8)
Supplement: Supplementary file 1 — Supplementary information [file 41598_2017_7373_MOESM1_ESM.pdf]

Nanosecond laser coloration on stainless steel surface

Yan Lu[1,2,*], Xinying Shi[2,*], Zhongjia Huang[3], Taohai Li[4], Meng Zhang[5], Jakub Czajkowski[6], Tapio Fabritius[6], Marko Huttula[2], and Wei Cao[2]

[1] School of Materials Science and Engineering, Henan University of Science and Technology, Luoyang 471023, China.

[2] Nano and Molecular Systems Research Unit, University of Oulu, P.O. Box 3000, FIN-90014, Oulu, Finland.

[3] School of Mechanical and Automotive Engineering, Anhui Polytechnic University, Wuhu 241000, China.

[4] College of Chemistry, Key Lab of Environment Friendly Chemistry and Application in Ministry of Education, Xiangtan University, Yuhu District, Xiangtan 411105, China.

[5] Department of Physics, East China University of Science and Technology, Meilong Road 130, Shanghai 200237, China.

[6] Optoelectronics and Measurement Techniques Research Unit, University of Oulu, P.O. Box 4500, FIN-90014, Oulu, Finland.

[*] These authors contributed equally to this work. Correspondence and requests for materials should be addressed to Y.L. (email: yan.lu@oulu.fi) or to W.C. (email: wei.cao@oulu.fi).

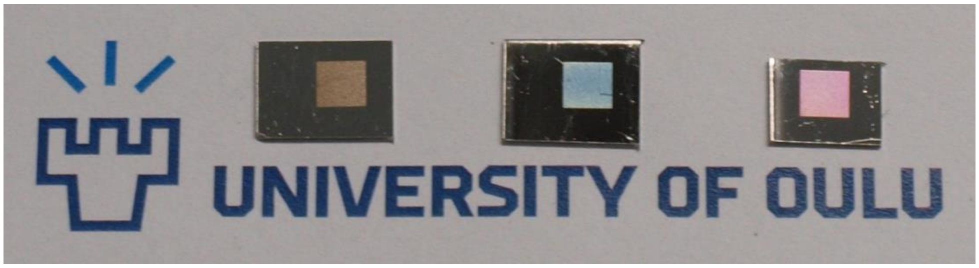

Supplementary Figure 1. Photograph of the samples. Three laser markings with different colors were prepared on stainless steel surface. The samples were fixed to a white A4 paper at a tilted direction, and then photographed by an Olympus digital camera under the irradiation of daylight lamp.

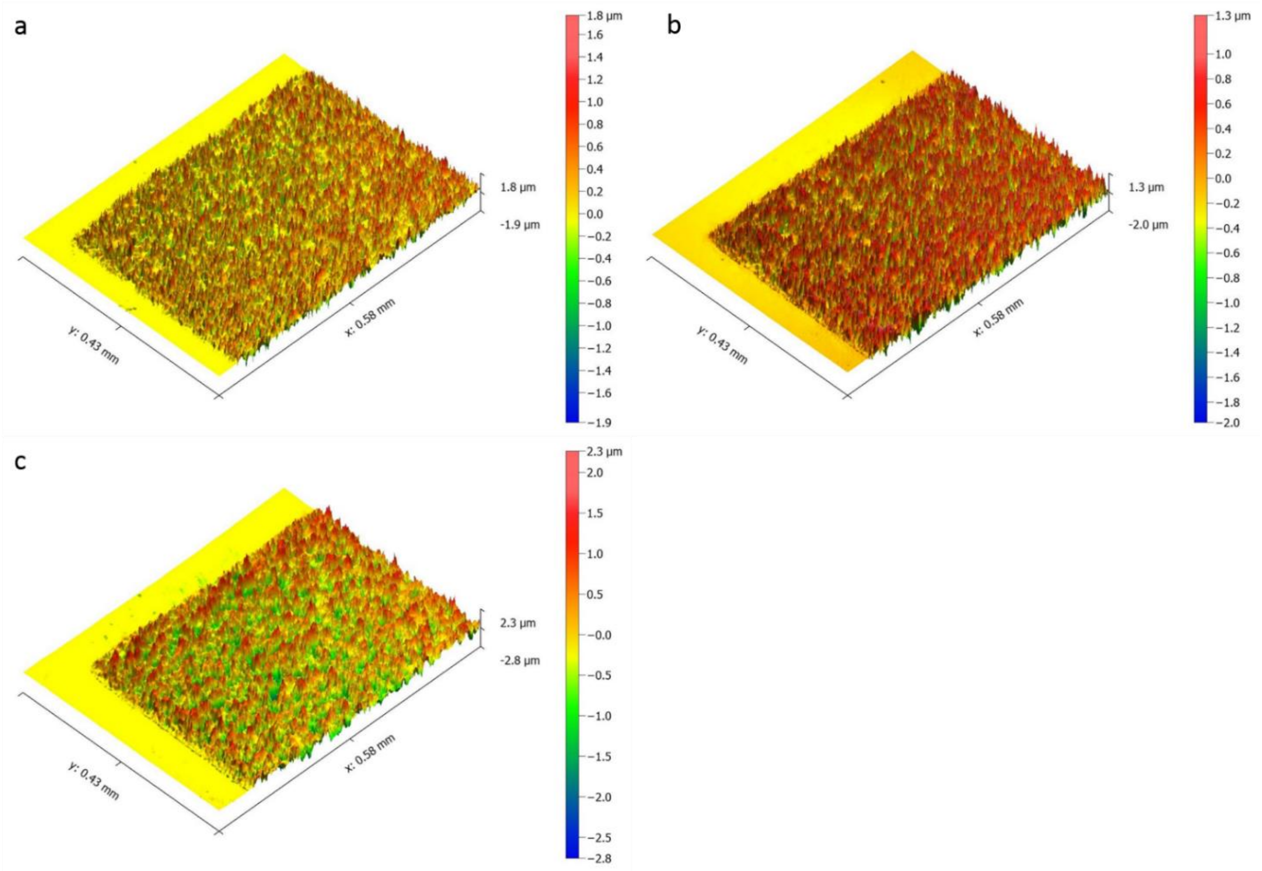

Supplementary Figure 2. Surface profile of the samples: (a) Sample 1, (b) Sample 2, (c) Sample 3. The profile images illustrate the surface roughness and morphology, and the thickness of laser markings can be estimated by the color scales.

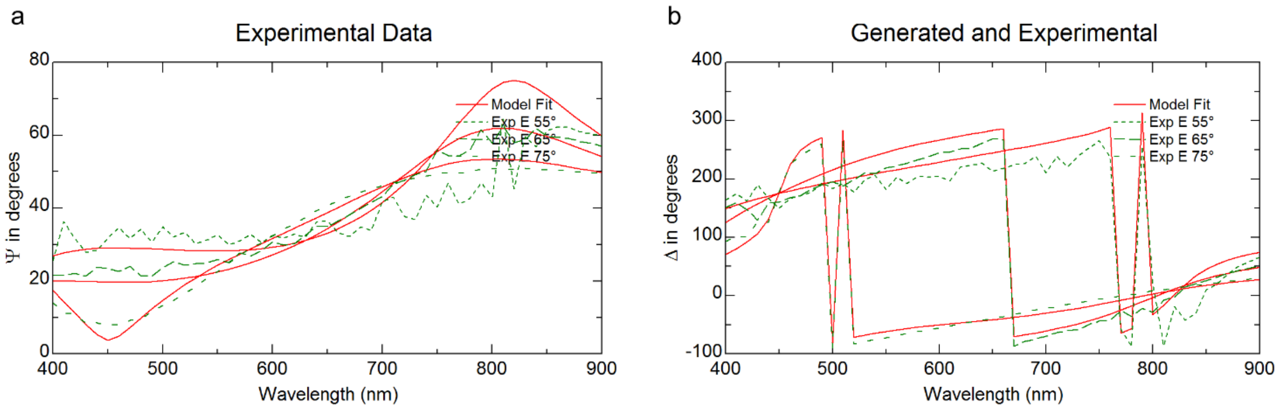

c

	Model	
1	Cauchy	314.400 nm
0	Stainless steel substrate	1 mm

Supplementary Figure 3. Ellipsometry measurement results. (a) Polarization orientation psi (Ψ), (b) Polarization phase delta (Δ), (c) Measured results based on the model established with Ψ and Δ values.

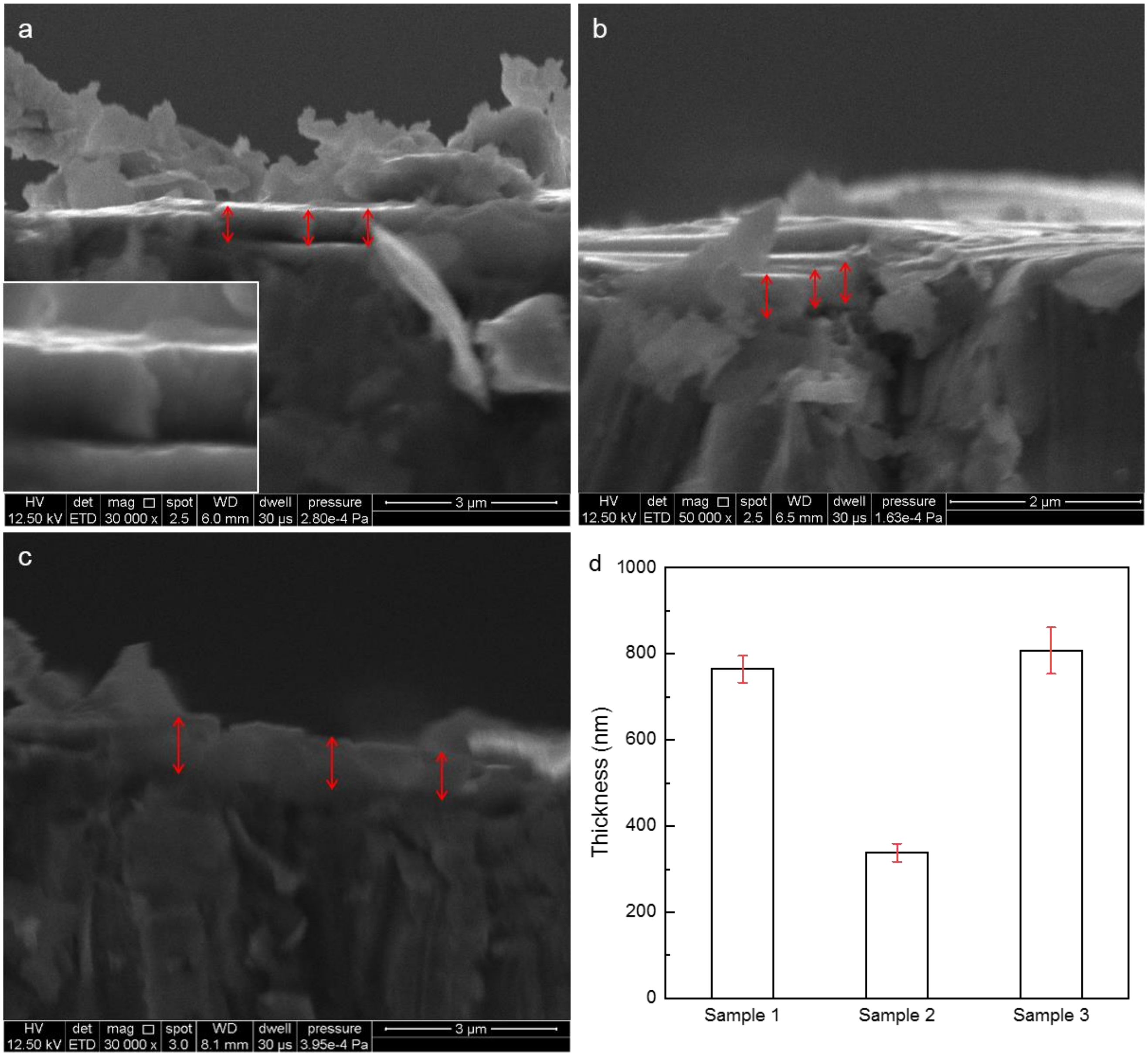

Supplementary Figure 4. Cross-sectional SEM images of samples: (a) Sample 1, (b) Sample 2, and (c) Sample 3, (d) Average thickness of sample 1 – 3. A zoomed-in region of sample 1 is shown in the inset. The arrow marks show the positions where thickness measurements were taken.

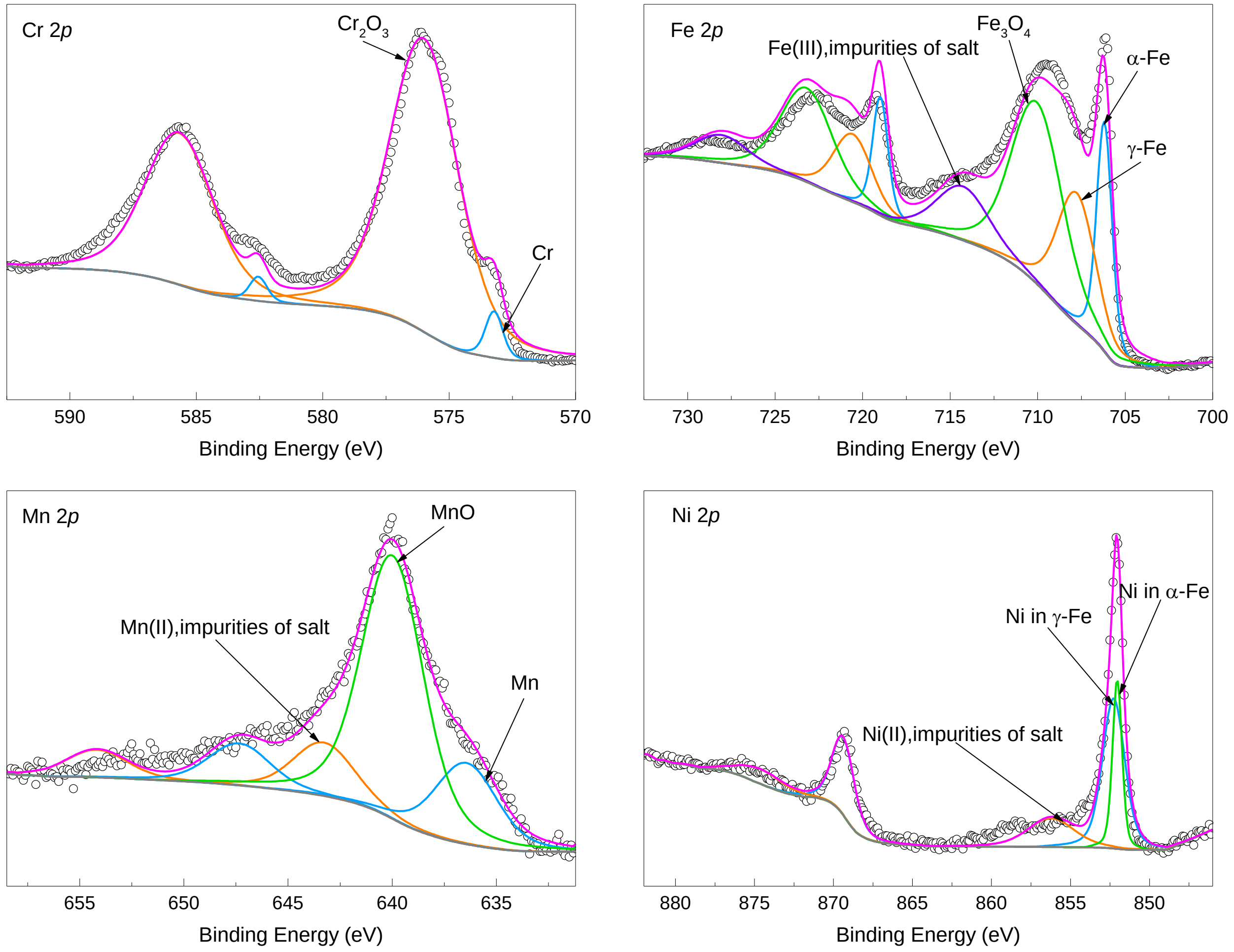

Supplementary Figure 5. XPS spectra of Cr 2*p*, Fe 2*p*, Mn 2*p* and Ni 2*p* of stainless steel. The peak of metal form were identified in all the spectra. Impurity peaks of Fe (III), Mn (II) and Ni (II) was specified as their salt compounds. They are common compositions for stainless steel. Each pair of doublet peaks were illustrated with identical color.

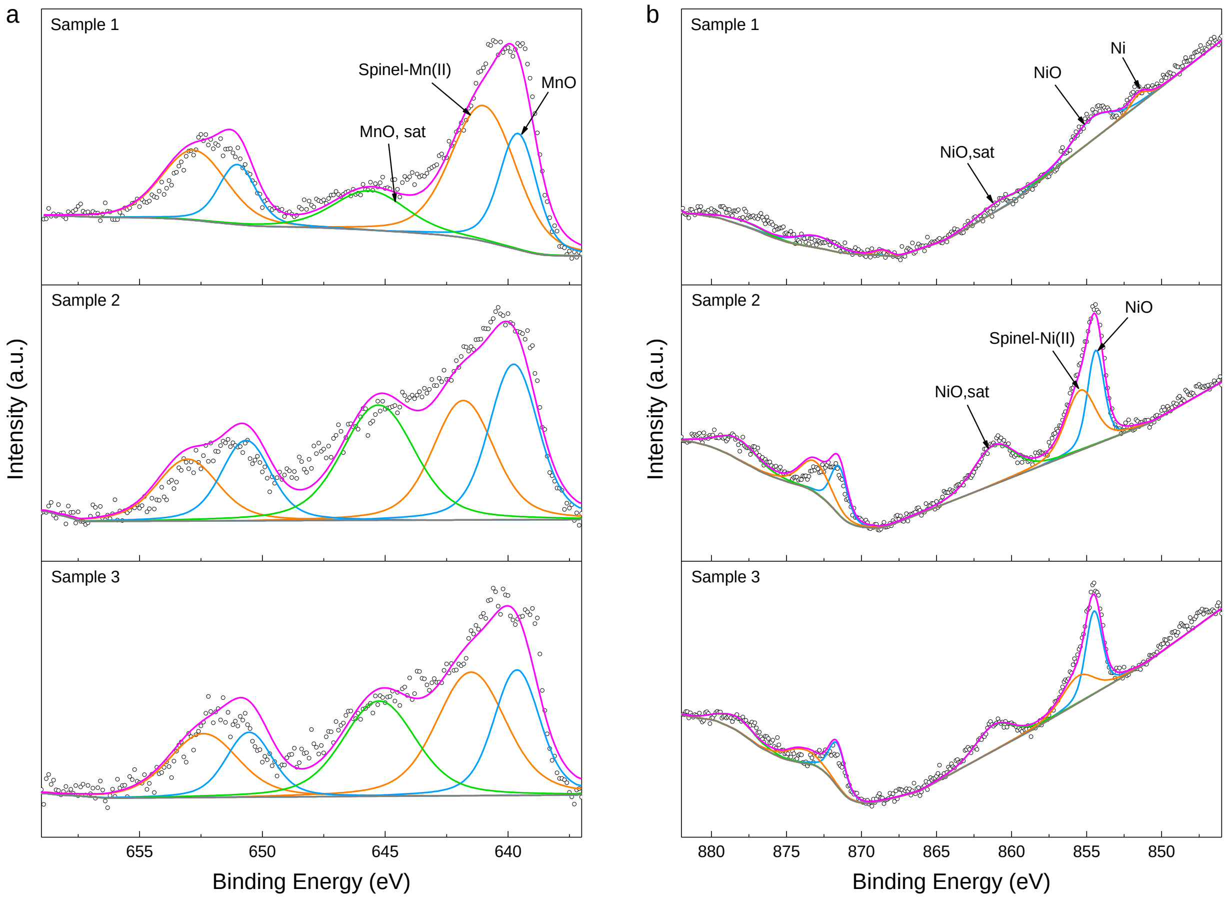

Supplementary Figure 6. XPS spectra of (a) Mn 2*p*, (b) Ni 2*p*. The scatter plots are experimental results, while the magenta and grey lines represent the fitting envelopes and backgrounds, respectively.

Supplementary Table 1. Elemental contents obtained by EDS. All values are in atomic percentage.

	Cr (%)	Fe (%)	Ni (%)	Mn (%)
Sample 1	17.80	65.14	6.75	1.27
Sample 2	13.50	60.86	6.76	0.78
Sample 3	10.36	52.25	6.04	0.66

Supplementary Table 2. Peak fitting details of Cr 2*p*, Fe 2*p*, Mn 2*p* and Ni 2*p*.

Valence states	Samples	Components	Binding Energy (eV)	FWHM (eV)	Atomic Percent (%)
Cr 2p	Sample1	spinel-Cr(III)	575.54	1.91	17.67
		Cr_2O_3	576.65	2.97	20.89
	Sample2	spinel-Cr(III)	575.77	1.90	3.57
		Cr_2O_3	576.91	3.00	3.31
	Sample3	spinel-Cr(III)	575.83	1.85	4.02
		Cr_2O_3	576.92	3.00	3.43
Fe 2p	Sample1	spinel-Fe(II,III)	709.11	3.34	32.39
		Fe_2O_3	711.84	3.50	15.69
		spinel-Fe(II,III), sat	715.75	3.50	8.08
	Sample2	spinel-Fe(II,III)	709.25	3.19	42.79
		Fe_2O_3	711.76	3.50	30.29
		spinel-Fe(II,III), sat	715.96	3.50	10.79
	Sample3	spinel-Fe(II,III)	709.32	3.08	43.07
		Fe_2O_3	711.74	3.50	32.33
		spinel-Fe(II,III), sat	716.25	3.50	11.15
Ni 2p	Sample1	Ni	851.49	1.31	0.12
		NiO	854.66	3.11	0.63
		NiO, sat	860.82	3.50	0.28
	Sample2	NiO	854.39	1.31	1.64
		spinel-Ni(II)	855.41	2.34	1.88
		NiO, sat	861.17	3.30	1.71
	Sample3	NiO	854.51	1.31	1.25
		Spinel-Ni(II)	855.56	2.52	0.79
		NiO, sat	861.18	2.96	0.88
Mn 2p	Sample1	MnO	639.59	1.80	1.25
		spinel-Mn(II)	640.99	3.12	2.45
		MnO, sat	645.63	3.50	0.54
	Sample2	MnO	639.77	2.38	1.51
		spinel-Mn(II)	641.82	2.93	1.43
		MnO, sat	645.28	3.50	1.08
	Sample3	MnO	639.64	2.23	0.94
		spinel-Mn(II)	641.50	3.34	1.39
		MnO, sat	645.24	3.50	0.74
